# Predicting the potential geographical distribution of onion thrips, *Thrips tabaci* in India based on climate change projections using MaxEnt

**DOI:** 10.1038/s41598-023-35012-y

**Published:** 2023-05-16

**Authors:** V. Karuppaiah, R. Maruthadurai, Bappa Das, P. S. Soumia, Ankush S. Gadge, A. Thangasamy, S. V. Ramesh, Dhananjay V. Shirsat, Vijay Mahajan, Hare Krishna, Major Singh

**Affiliations:** 1 ICAR-Directorate of Onion and Garlic Research, Rajgurunagar, Pune, Maharashtra 410 505 India; 2 ICAR-Central Coastal Agricultural Research Institute, Ela, Old Goa, Goa, 403 402 India; 3 ICAR-Central Plantation Crops Research Institute, Kasaragod, Kerala 671 124 India; 4 ICAR-Indian Institute of Vegetable Research, Varanasi, Uttar Pradesh 221 305 India

**Keywords:** Behavioural ecology, Biogeography, Climate-change ecology, Ecological modelling, Population dynamics, Entomology

## Abstract

Onion thrips, *Thrips tabaci* Lindeman, an economically important onion pest in India, poses a severe threat to the domestic and export supply of onions. Therefore, it is important to study the distribution of this pest in order to assess the possible crop loss, which it may inflict if not managed in time. In this study, MaxEnt was used to analyze the potential distribution of *T. tabaci* in India and predict the changes in the suitable areas for onion thrips under two scenarios, SSP126 and SSP585. The area under the receiver operating characteristic curve values of 0.993 and 0.989 for training and testing demonstrated excellent model accuracy. The true skill statistic value of 0.944 and 0.921, and the continuous Boyce index of 0.964 and 0.889 for training and testing, also showed higher model accuracy. Annual Mean Temperature (bio1), Annual Precipitation (bio12) and Precipitation Seasonality (bio15) are the main variables that determined the potential distribution of *T. tabaci,* with the suitable range of 22–28 °C; 300–1000 mm and 70–160, respectively. *T. tabaci* is distributed mainly in India's central and southern states, with 1.17 × 10^6^ km^2^, covering 36.4% of land area under the current scenario. Multimodal ensembles show that under a low emission scenario (SSP126), low, moderate and optimum suitable areas of *T. tabaci* is likely to increase, while highly suitable areas would decrease by 17.4% in 2050 20.9% in 2070. Whereas, under the high emission scenario (SSP585), the high suitability is likely to contract by 24.2% and 51.7% for 2050 and 2070, respectively. According to the prediction of the BCC-CSM2-MR, CanESM5, CNRM-CM6-1 and MIROC6 model, the highly suitable area for *T. tabaci* would likely contract under both SSP126 and SSP585. This study detailed the potential future habitable area for *T. tabaci* in India, which could help monitor and devise efficient management strategies for this destructive pest.

## Introduction

Climate change is the major threat to global food security, health, livelihoods, and economy^1,2^. Climate change affects the insect species' abundance, developmental cycle, voltinism, dispersal, migration, distribution pattern, pest invasion and outbreak, habitat suitability and host expansion, and adaptability to a broader range of biogeographic conditions^3–6^. In the coming century, the earth may warm by 1.4–5.8 °C, according to global climate models^7^. The increase in global average temperature and atmospheric CO_2_, erratic rainfall patterns, prolonged droughts, floods, and an increased risk or incidence of pests and diseases all indicate climate change's adverse impacts on agriculture^8,9^.

Onion thrips, *Thrips tabaci* (Thysanoptera: Thripidae) is a belligerent pest of onion reported globally^10^. Karl Eduard Lindeman, a Russian entomologist, initially identified the pest as *T. tabaci*^11^. Initially, the species was reported to be present in the eastern Mediterranean region later it gradually spread throughout the world^12^. Globally, annual crop loss due to onion thrips is estimated to be over 1 billion US$^13^. *Thrips tabaci* is regarded as a pest of national significance^14^ in India and its feeding damage results in an annual yield loss of 10–15% in the onion crop^15^. Thus, *T. tabaci* is one of the major threats to Indian onion industry, affecting the domestic supply and processing of the crop as well as denting the annual foreign exchange earnings estimated worth of 377.8 million US$ in 2020–2021^16^. The pest attacks onions irrespective of the growing seasons but the intensity of damage varies depending on the season, local climatic conditions, and hosts. Both the nymphs and adults suck the plant sap, causing small white blister in the early stage of the attack, which later turns into a larger batch of the silvery blister, causing a significant reduction in photosynthesis^17^, leading to the undersized onion bulbs^18,19^. *Thrips tabaci*, in addition to causing leaf damage, also serves as a vector of destructive Iris yellow spot virus disease in onion^20^ and aggravates fungal diseases in onion^21,22^. The polyphagous nature, high reproductive rate, short life-cycle, asexual mode of reproduction and off-season survival pose major challenges in managing *T. tabaci* menace in onion^23^.

The occurrence, distribution, abundance, and developmental rate of onion thrips are all affected mainly by climatic variables, much like any other insect^24–27^. Generally, thrips populations multiply in hot and dry weather, while heavy rains wipe the thrips off the plants^28^. The water-deficit stress in plants affects plant nutrition and favors thrips attack^29^. The extreme temperature could arrest the development of insects whereas cool weather with moderate temperature favours the population build-up^26^. Temperature affects larval development in thrips, as temperature between 15 and 25 °C has been found to be optimum for maximum (> 80%) hatchability, and adult longevity decreases with increasing temperature.

Considering the impact of environmental factors on species distribution, dispersion, and abundance^30^, there is a concern that increasing temperature and CO_2_ rise could alter the distribution pattern of *T. tabaci* among the onion-growing regions of India. The projected increase of air temperature^31–33^ will accelerate *T. tabaci* development cycle and prolong the period of favourable climate, which may result in multiple generations in a crop season^25,27^. The changes observed in pest ecology are consistent with climate change predictions and their impacts^34^. In this context, the prediction of habitat suitability of a pest under changing climate using niche models could help identify potential risk areas that will facilitate in framing appropriate pest mitigation strategies aimed at management and arresting their spread into hitherto unsuitable areas.

Using pest occurrence records and associated bioclimatic variables, ecological niche modelling (ENM) has been successfully harnessed to evaluate the potential distribution and spread of pests^35–37^. A correlative species distribution model (SDM) called MaxEnt (maximum entropy modelling) has been extensively employed to assess the species distribution and suitable habitat for a multitude of cosmopolitan pests and invasive species^38–40^. The present study aims to determine how climate change may impact the distribution of suitable habitats for onion thrips, to map the current distribution in India and quantify the changes in risk of the pest under projected climate scenarios. It will enumerate the large-scale drivers of *T. tabaci* distribution and assist in identifying future hotspots for targeted control. This study will help determine imminent hotspots for focused management by listing the major factors that influence the distribution of *T. tabaci*.

## Materials and methods

### Occurrence data of *T. tabaci*

The occurrence data of *T. tabaci* were obtained from pest survey conducted from 2017 to 2021 under All India Network Research Project on Onion and Garlic for potential occurrence points among the different states of India and from published sources^15,41–45^. The species data were collected regardless of season and covered major onion-growing states of India. A total of 125 occurrence points representing all major onion-growing regions of India were used for the modeling. The locations of occurrence records are shown in Fig. 6a (Refer Supplementary file 1).

### Bioclimatic variables and analysis

Bioclimatic variables are biologically meaningful indicators that describe how climate affects ecosystems and services. They are derived from monthly temperature and rainfall values that then represent annual and seasonal climatic trends. Data with a spatial resolution of 2.5 arc-min (4.6 km resolution at the equator) and 19 bioclimatic variables retrieved from the WorldClim database (http://www.worldclim.org/) were utilized for the analysis (Supplementary table 2). Using the ‘ENMTools’ package in the R programming language, the cross-correlations among the bioclimatic variables were evaluated^46^. Multi-collinearity analysis was carried out among the predictor variables to exclude the causal variable and ensure that the model is statistically sound in its ability to explain variation in the response variable. The variables with correlation coefficient (|r|≥ 0.8; very significant correlation), that are biologically important for *T. tabaci* distribution were screened^47^. To determine the potential geographic distribution of *T. tabaci*, nine bioclimatic variables were selected such as the annual mean temperature (bio1), mean diurnal range (bio2), isothermality (bio3), temperature seasonality (bio4), annual precipitation (bio12), precipitation of the wettest month (bio13), precipitation of the driest month (bio14), precipitation seasonality (bio15), precipitation of the warmest quarter (bio18), and precipitation of the coldest quarter.

Data sets of bioclimatic factors for the current (1970–2000), 2050 (2041–2060), and 2070 (2061–2080) scenarios were used to determine the present and future potential distribution. New future trajectories based on socio-economic assumptions were built using Shared Socioeconomic Pathways (SSPs) reflecting various socio-economic growth levels^48^. Three types of SSPs are categorized: SSP126 for low-forcing scenarios, SSP245 for medium-forcing scenarios and SSP585 for high-forcing scenarios. To represent low and high emission scenarios, respectively, the SSP126 and SSP585 were used. In order to fit these two new scenarios, future climate data for the years 2050 and 2070 were downscaled from the BCC-CSM2-MR (Beijing Climate Center Climate System Model), CNRM-CM6-1 (Centre National de Recherches Meteorologiques, Centre Europeen de Recherche et de Formation Avancee en CalculScientifque), canESM5 (Canadian Earth System Model 5), and MIROC6 (Model for Interdisciplinary Research on Climate) from the CMIP6 of the sixth assessment report (AR6) of the Intergovernmental Panel on Climate Change (IPCC).

### MaxEnt modeling

The distribution of *T. tabaci* was predicted using MaxEnt (maximum entropy species distribution modelling), version 3.4.0, which is accessible at http://biodiversityinformatics.amnh.org/opensource/maxent/. The feature classes (FCs) and regularization multipliers (RMs) are parameters that impact the MaxEnt model’s complexity. The overfitting of the model is managed by the RMs. The original data set of environmental variables are transformed into FCs, particularly linear (L), quadratic (Q), product (P), threshold (T), and hinge (H)^49,50^. In the current study, all possible combinations of FCs taking one, two, three, four and five features at a time with RMs varying between 0.5 and 5 with an interval of 0.5 were tested using “ENMEval” package^51^ in R statistical software Version 4.2.0^52^. Optimizing FC and RM was carried out using tenfold random cross-validation of the total occurrence dataset. Akaike’s Information Criterion with small sample size correction (AICc) was used for selection of optimum MaxEnt model parameters. The model parameter combination with the smallest AICc (delta.AICc = 0) was used for further analysis. The final settings for the MaxEnt model were as follows: maximum iterations = 5000, convergence threshold = 0.0001, maximum number of background points = 10,000, format of the model output = Cloglog, random test percentage = 25, regularization multiplier = 2, feature classes = linear, hinge and threshold features (LHT). The MaxEnt model is a popular method for forecasting a species' geographic distribution with presence-only data, and it still exhibits good performance with small sample sizes^53,54^.

### Model fitting and evaluation

Nine bio-climatic factors, which are significant distributional drivers, were utilized to run the model, and 125 sites with *T. tabaci* presence-only data were examined. *Thrips tabaci* occurrence data was randomly split into two quasi-independent subsets^55^, each containing 75% and 25% of the data for the model's training and testing, respectively. The average values of the area under the curve (AUC) represent the significance of the factors influencing *T. tabaci*. To quantify the errors and assess the consistency of the model, the model was also fitted on the data set using tenfold cross-validation^56^. To measure the model’s accuracy, the area under the receiver operating characteristic (ROC) curve was selected^57,58^. The continuous Boyce index (CBI) and the true skill statistic (TSS) were also used for model evaluation. TSS was calculated using the maximum training sensitivity plus specificity Cloglog threshold.

The Jackknife test was performed to measure a variable with high importance in predicting the potential species distribution. To predict the suitability of future habitat of *T. tabaci*, the output of the MaxEnt model was further projected onto a spatial map for the chosen climate change scenarios (SSP126 and SSP585) downscaled from BCCCSM2-MR, CNRM-CM6-1, canCSM5, and MIROC6. ArcGIS 9.1 software was used for spatial mapping in order to create maps of suitability for both present and future climate change scenarios. The distribution of *T. tabaci* in the future was extrapolated from the base map of India. On the map, degrees of habitat suitability were classified into five classes based on ‘maximum training sensitivity plus specificity Cloglog threshold’ as the high habitat suitability area (0.778–1), the optimum habitat suitability (0.584–0.778), the medium habitat suitability (0.389–0.584), the low habitat suitability (0.195–0.389), and the unsuitable habitat (0.0–0.195)^5^.

## Results

### Model performance and validation

**Figure 1:**
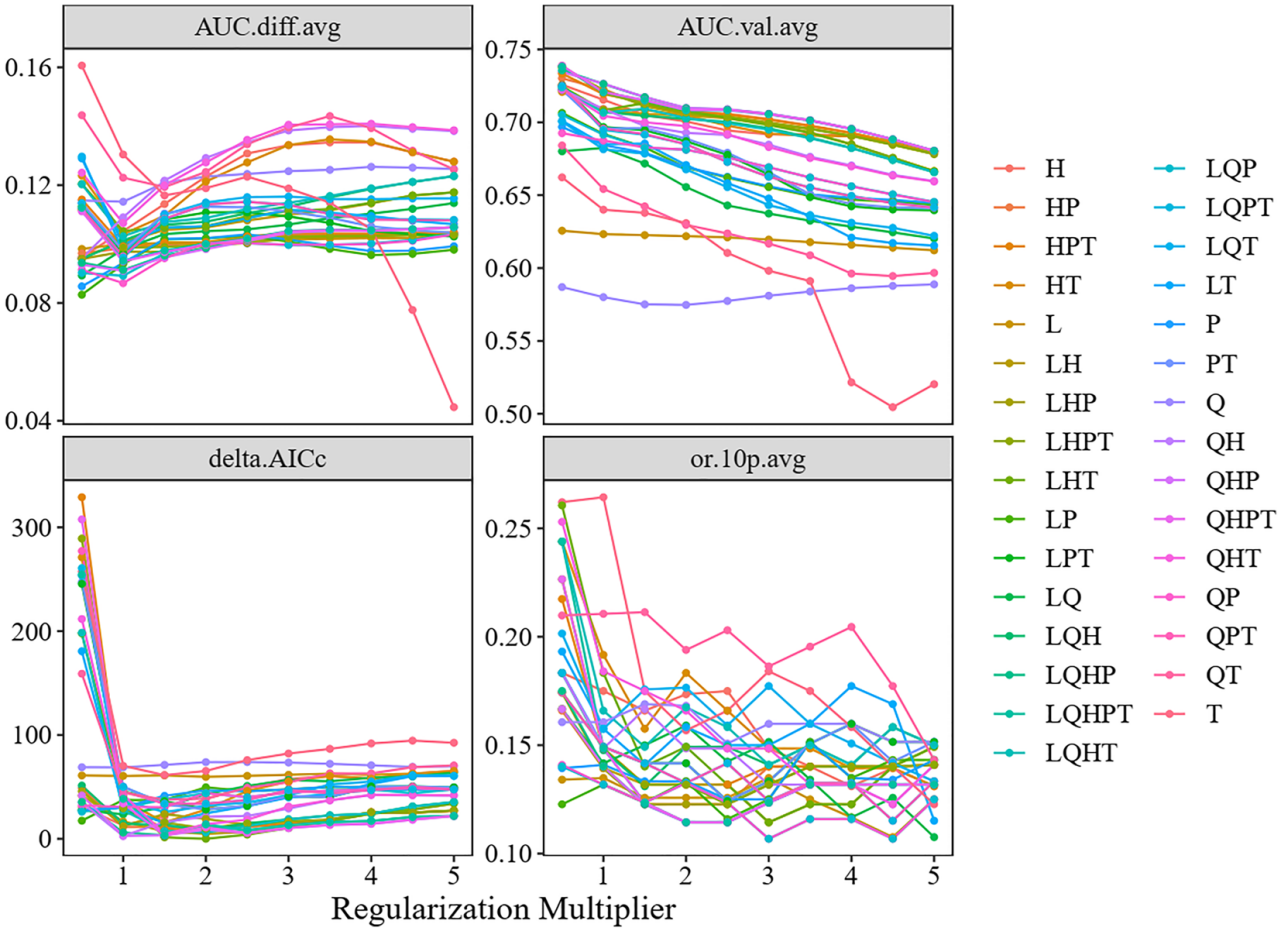
The output of MaxEnt models optimization using different combinations of regularization multipliers and feature classes.

**Figure 2:**
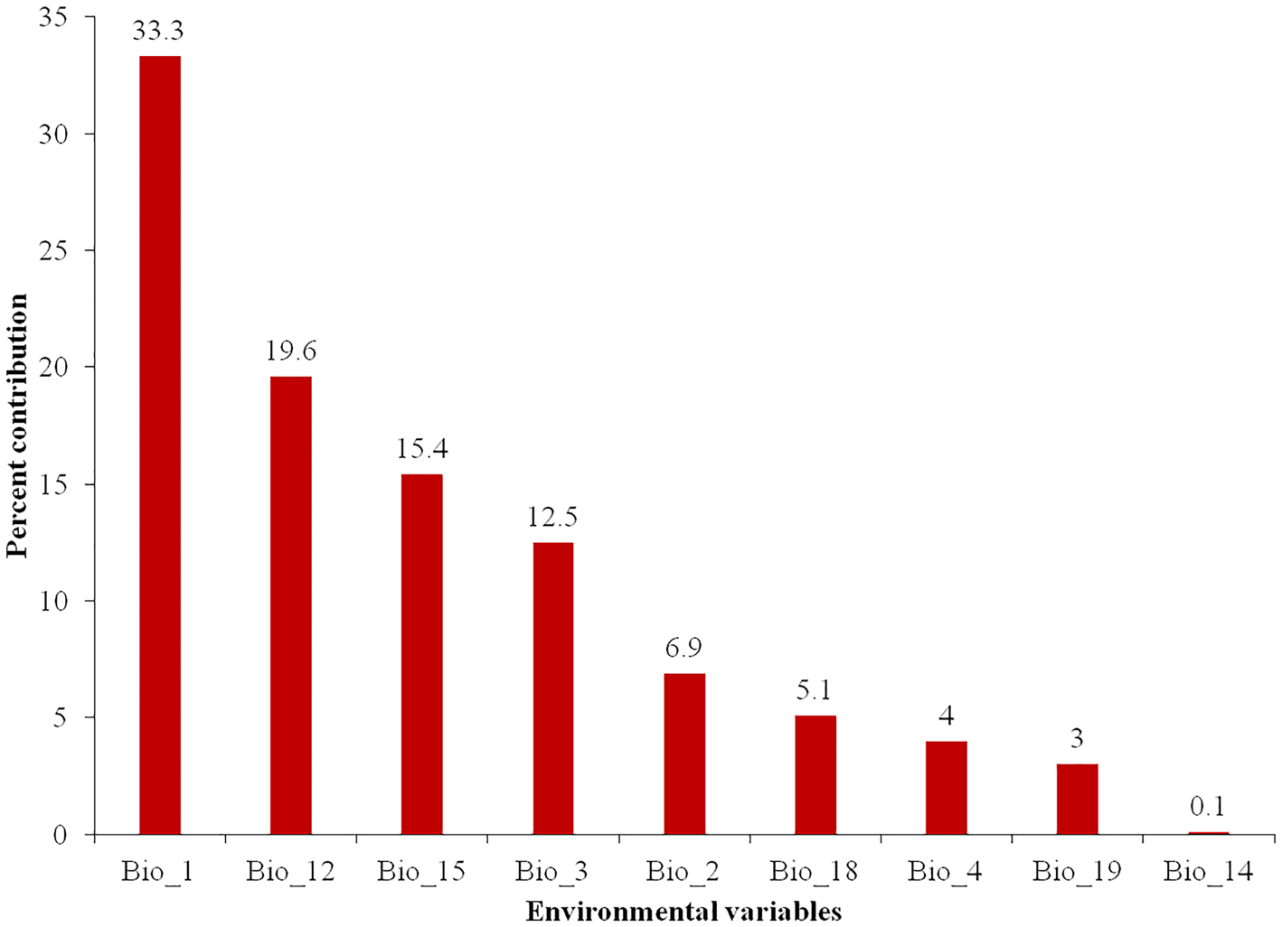
Relative contribution of bioclimatic variable to MaxEnt model for *T. tabaci*.

The output matrices of feature class (FC) and regularization multiplier (RM) combinations are depicted in Fig. 1. The best FC combination had linear, hinge and threshold features (LHT) with RM of 1 (delta.AICc = 0, AICc = 2647.177, AUC.val.avg = 0.708, AUC.diff.av = 0.106, or.10P.avg = 0.19) and was based on the delta.AICc. The MaxEnt model predicted that throughout the 10 cross-validation iterations, the average testing AUC and training AUC were 0.990 and 0.992, respectively, and the TSS and CBI for the test and training were 0.820 and 0.973, respectively (Table 1). The higher average training AUC and a test AUC value suggested high predictive accuracy of the MaxEnt model, which performs better than random models in predicting the potential distribution of *T. tabaci.* The relative contribution of each bioclimatic variable in predicting potential species distribution shows that the Annual Mean Temperature (bio1; 33.3%) was the foremost important environmental variable in defining the insects’ potential distribution followed by Annual Precipitation (bio12; 19.6%) and Precipitation Seasonality (bio15; 15.4%) (Fig. 2). All these factors together contributed 68.3% and found to be strongest predictors for *T. tabaci* distribution in India. The variables including Isothermality (bio3), Mean Diurnal Range (bio2), Precipitation of the Warmest Quarter (bio18), Temperature Seasonality (bio4), Precipitation of the Coldest Quarter (bio19), and Precipitation of the Driest Month (bio14), also contributed 12.5%, 6.9%, 5.1%, 4.0%, 3.0%, and 0.1%, respectively.

**Table 1:** Evaluation statistics i.e., value for area under the curve (AUC), true skill statistic (TSS), continuous Boyce index (CBI) values of ten-fold cross-validation and random sampling using training and test dataset.

Replicates and data partitioning	Training			Test		
	AUC	TSS	CBI	AUC	TSS	CBI
Replication 1	0.992	0.954	0.987	0.993	0.797	0.959
Replication 2	0.992	0.943	0.982	0.993	0.962	0.961
Replication 3	0.992	0.958	0.979	0.994	0.894	0.982
Replication 4	0.993	0.944	0.954	0.989	0.963	0.444
Replication 5	0.992	0.946	0.986	0.992	0.818	0.894
Replication 6	0.992	0.959	0.960	0.992	0.969	0.873
Replication 7	0.993	0.958	0.958	0.981	0.968	0.887
Replication 8	0.992	0.959	0.976	0.990	0.887	0.654
Replication 9	0.992	0.957	0.965	0.994	0.967	0.708
Replication 10	0.993	0.953	0.980	0.976	0.963	0.842
Average	0.992	0.953	0.973	0.990	0.919	0.820
Random sampling (75:25)	0.993	0.944	0.964	0.989	0.921	0.889

**Figure 3:**
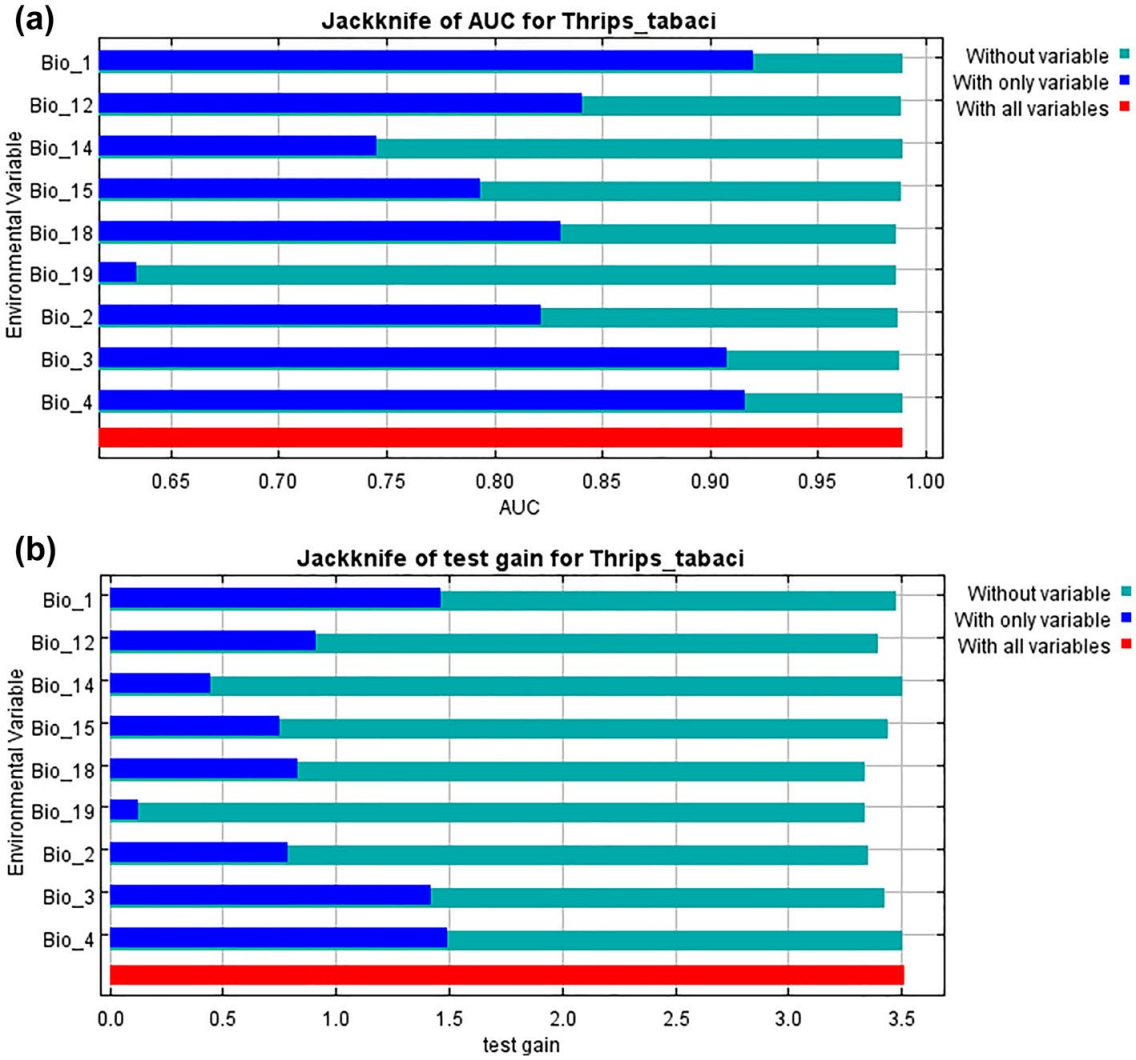
Relative importance of bioclimatic variables based on Jackknife test in MaxEnt. Horizontal bar shows the contribution of each variables to (**a**) Area under the (AUC) receiver operating characteristic curve (ROC) and (**b**) regularized test gain.

**Figure 4:**
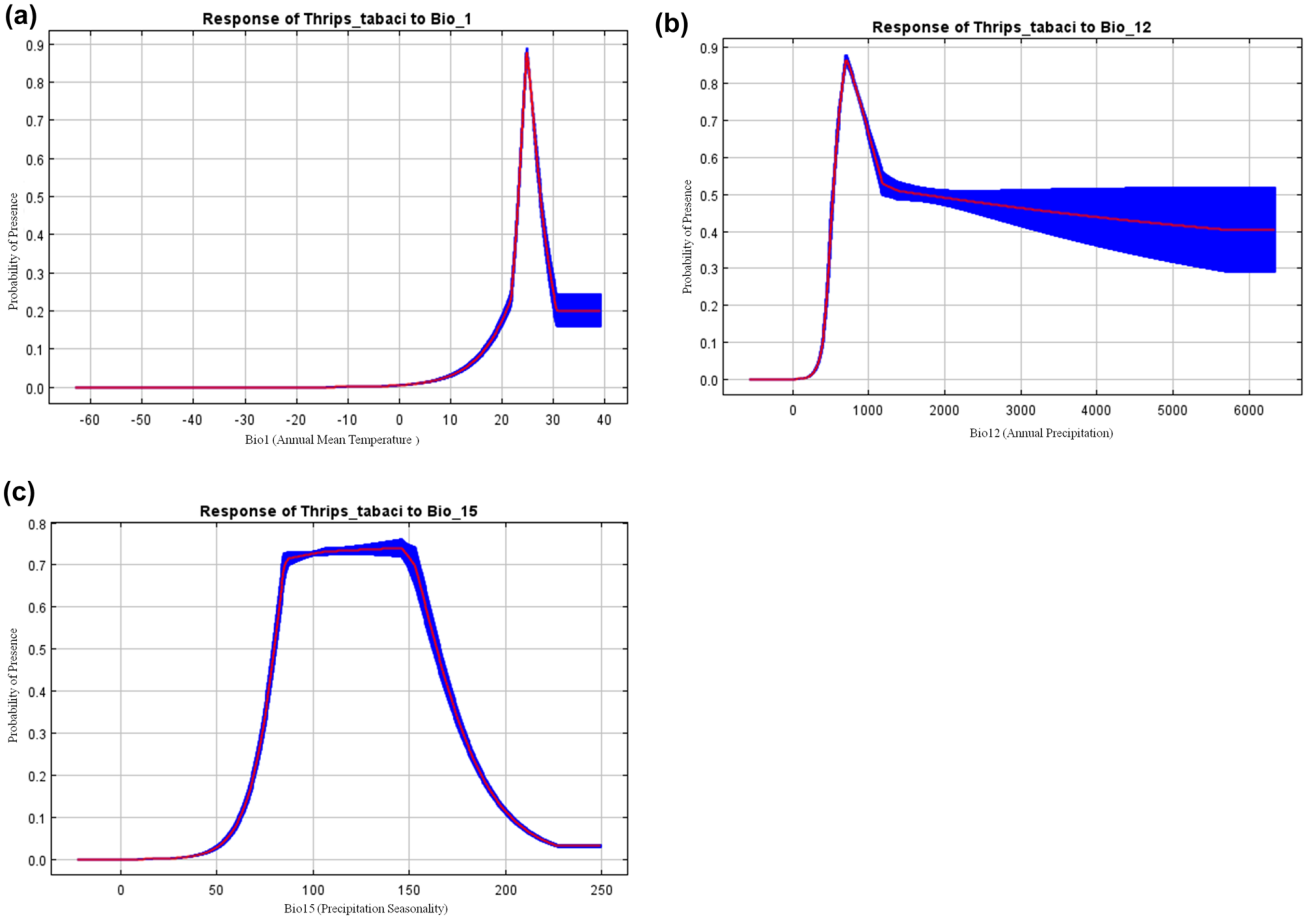
Response curves of top three environmental variables determining the *T. tabaci* distribution (**a**) bio1, (**b**) bio12 and (**c**) bio15. The red lines represent the mean, while the blue borders represent the SD for 10 replications.

Jackknife test revealed that Annual Mean Temperature (bio1) followed by Temperature Seasonality (bio4) and Isothermality (bio3) as the most influencing predictors, with high regularized training gain (Fig. 3). The likelihood of *T. tabaci* occurrence based on each factor's response curve for the major bioclimatic parameters depicted in Fig. 4. The probability of *T. tabaci* occurrence increased with the rise in Annual Mean Temperature (bio1) from 20.0 to 25.0 °C, and subsequently showed a decrease until 30 °C, before becoming steady between 30 and 40 °C. Likewise, the probability of species occurrence shows a negative correlation with increasing Annual Precipitation (bio12), where the species presence increased until 800 mm Annual Precipitation; after that, it showed a sharp downward. The curve of seasonal Precipitation revealed that the probability of occurrence increased from 50, was maximum at 80, then remained constant until 160, and after that exhibited a sharp decline.

### Potential current geographical distribution of *T. tabaci* in India under CMIP6 projection

The potential geographical distribution of the onion thrips' current habitat is shown in Fig. 6b. The MaxEnt predicted that 1.17 × 10^6^ km^2^ (36.4%) of India's total land area is highly suitable for *T. tabaci* establishment (Table 2). The highly suitable habitable areas in central India include the states of Maharashtra, Karnataka, Madhya Pradesh, Gujarat, and parts of Rajasthan. In the North, the parts of Uttar Pradesh, Bihar, Jharkhand, Chhattisgarh, Delhi, Haryana, Punjab and Uttarakhand and parts of northeastern states. Karnataka, Andhra Pradesh, Telangana and Tamil Nadu are highly suitable areas in southern India. The area under optimum suitability category was 7.44 × 10^5^ km^2^ which is 23.1% of total land area (Table 2). Approximately, 3.79 × 10^5^ km^2^ accounting for 11. 8% of land area calculated as moderately suitable and 1.95 × 10^5^ km^2^ (6.10%) as low suitable. Some areas that were low and moderately suitable at the current habitat were highly or optimally suitable for the future potential distribution of *T. tabaci*, specifically at higher latitudes.

**Table 2:** Current and future potential habitat predicted for *T. tabaci* under low (SSP126) and high (SSP585) emissions scenario (km^2^).

Suitability class	Present	SSP126 (2050)	SSP126 (2070)	SSP585 (2050)	SSP585(2070)
Unsuitable (0.0–0.195	7.39 × 10^5^	7.40 × 10^5^	7.37 × 10^5^	7.38 × 10^5^	7.58 × 10^5^
Low (0.195–0.389)	1.95 × 10^5^	2.75 × 10^5^	2.74 × 10^5^	3.07 × 10^5^	3.67 × 10^5^
Moderately (0.389–0.584)	3.79 × 10^5^	4.44 × 10^5^	4.78 × 10^5^	4.85 × 10^5^	5.80 × 10^5^
Optimum (0.584–0.778)	7.44 × 10^5^	7.71 × 10^5^	7.70 × 10^5^	7.54 × 10^5^	7.54 × 10^5^
Highly (0.778–1)	1.17 × 10^6^	1.00 × 10^6^	9.73 × 10^5^	9.47 × 10^5^	7.73 × 10^5^

### Potential future geographical distribution of *T. tabaci* under CMPI6 projection

**Figure 5:**
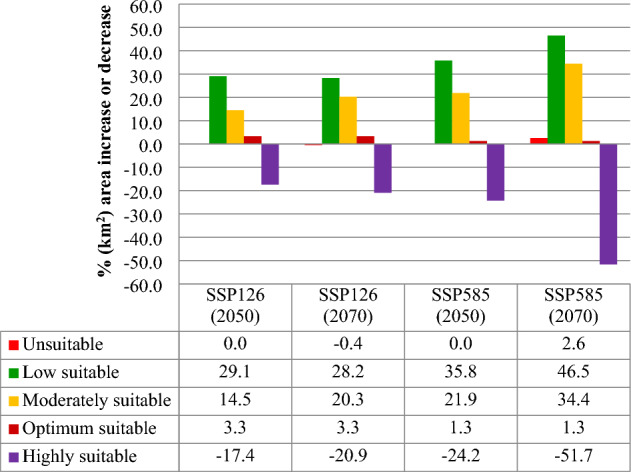
Estimated proportionate changes in the future potential habitat suitability from the current potential distribution.

The MaxEnt prediction for the scenarios SSP126 and SSP585 for the 2050s and 2070s are depicted in Figs. 7a-d, and 8a–d. Under SSP126, 77.3% (2.49 × 10^6^ km^2^) of India's total area was predicted to be suitable habitat in 2050 and 2070. The highly, optimum, moderately and low suitable habitable area under SSP126 (2050) was 1.00 × 10^6^ km^2^, 7.71 × 10^5^ km^2^, 4.44 × 10^5^ km^2^ and 2.75 × 10^5^ km^2^, respectively (Table 2). The model showed that 29.1%, 14.5%, and 3.3% increase in low, moderate, and optimum suitable habitat areas (Fig. 5). Furthermore, a reduction of 17.4% areas among the regions which are highly suitable under the current situations was projected under SSP126 (2050).

**Figure 6:**
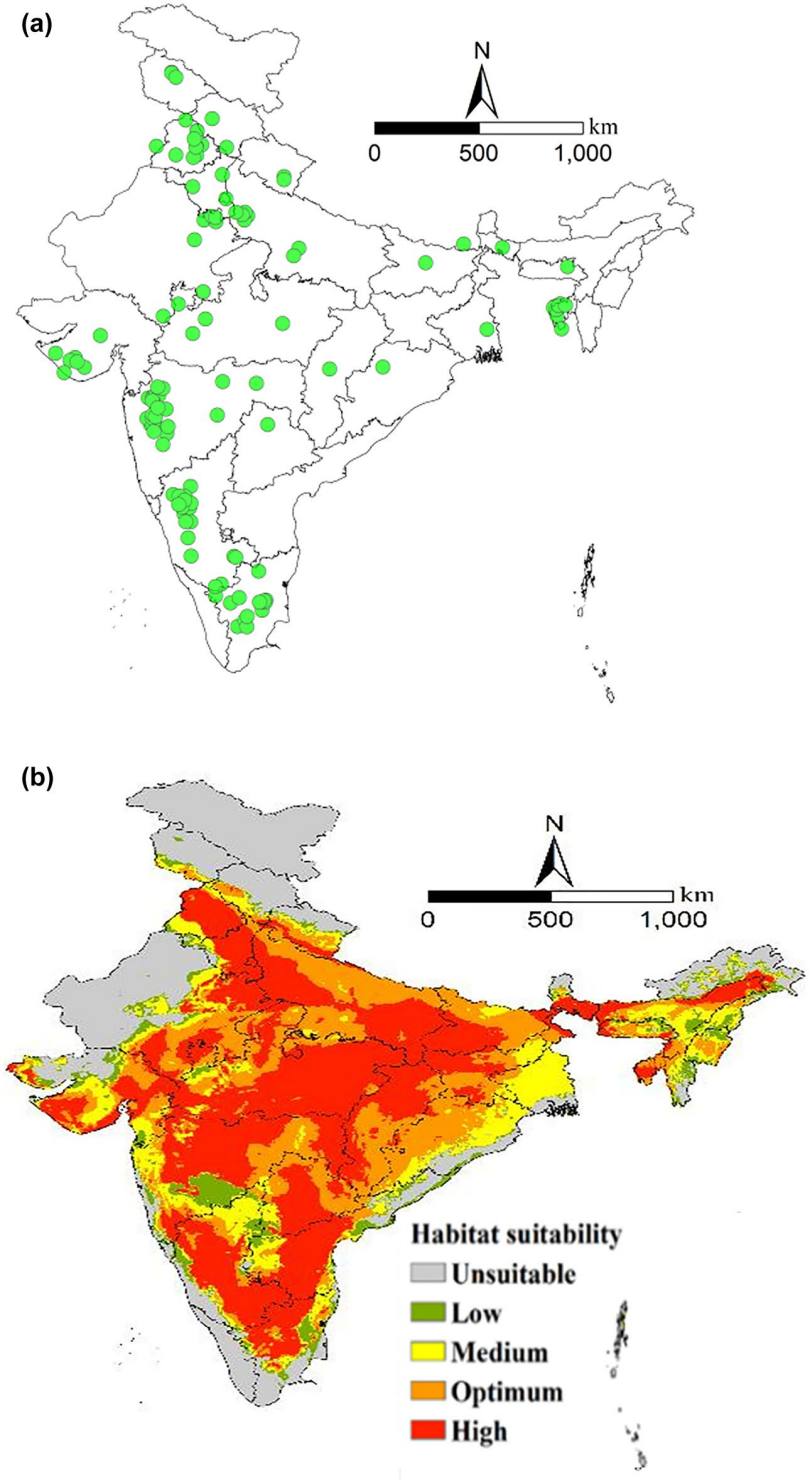
(**a**) *Thrips tabaci* occurrence points (**b**) Potential geographical distribution of *T. tabaci* in India under present conditions.

**Figure 7:**
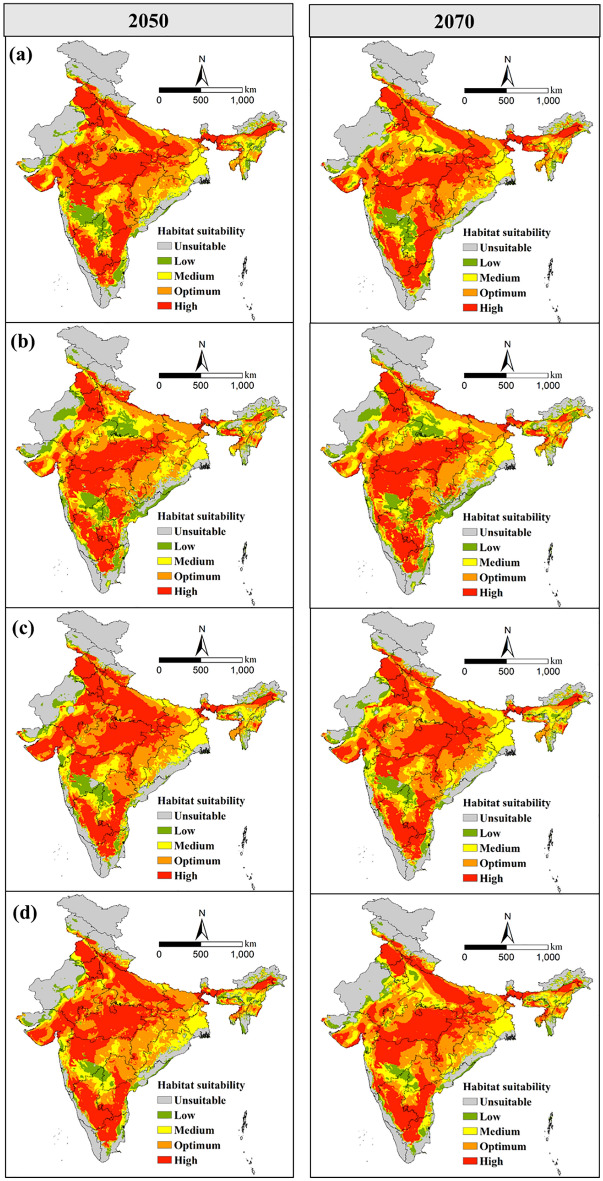
Potential future geographical distribution of *T. tabaci* in India based on (**a**) BCC-CSM2-MR, (**b**) CanESM5, (**c**) CNRM-CM6-1, (**d**) MIROC6 models for SSP126 during 2050 and 2070.

**Figure 8:**
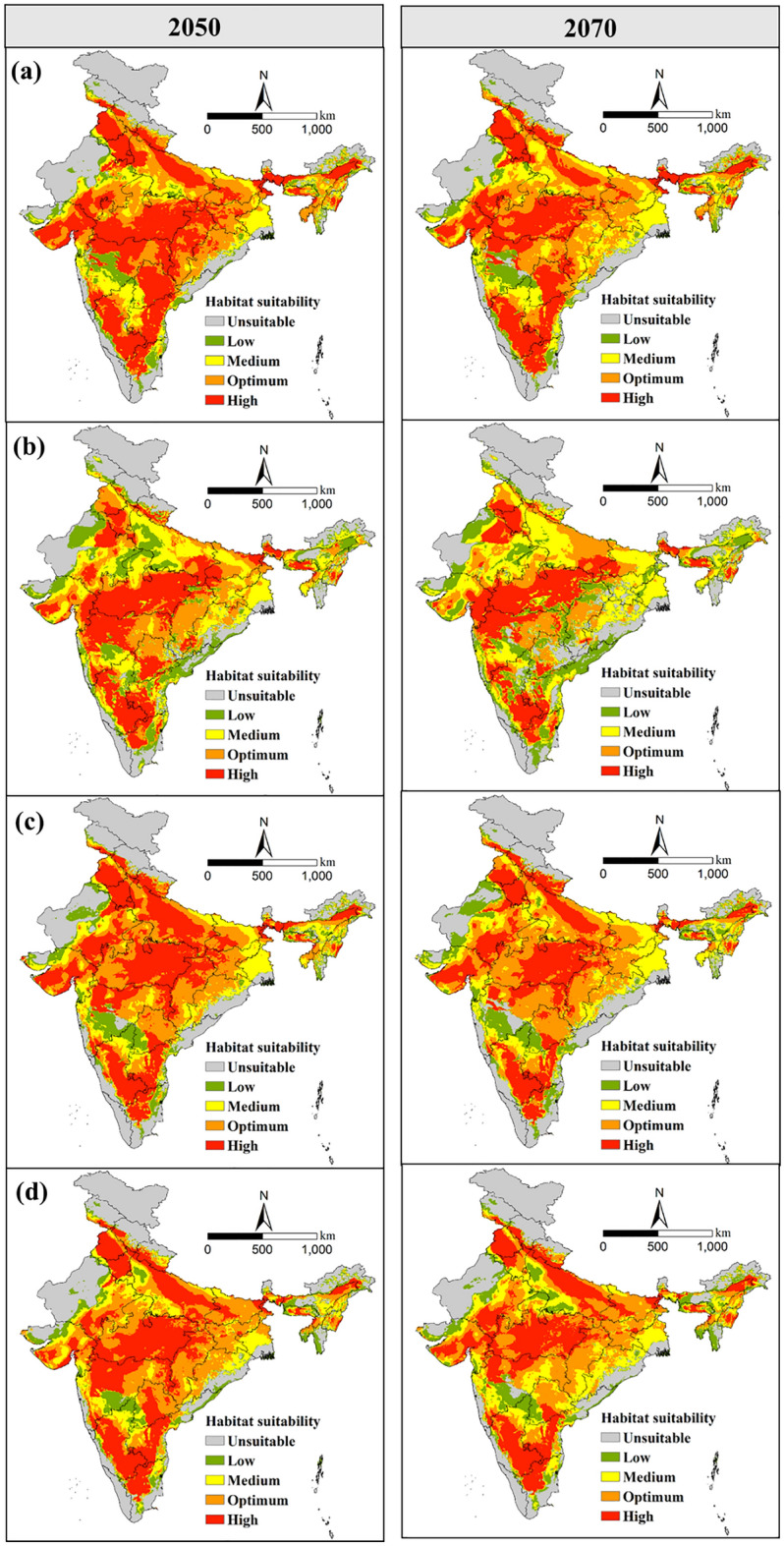
Potential future geographical distribution of *T. tabaci* in India based on (**a**) BCC-CSM2-MR, (**b**) CanESM5, (**c**) CNRM-CM6-1, (**d**) MIROC6 models for SSP585 during 2050 and 2070.

The highly, optimum, moderately and low suitable areas for 2070 under SSP126 was 9.73 × 10^5^ km^2^, 7.70 × 10^5^ km^2^, 4.78 × 10^5^ km^2^ and 2.74 × 10^5^ km^2^, respectively. The model projected a 28.2% increase in a low suitable area and a 0.4% decrease in unsuitable habitats for *T. tabaci* distribution compared to the current habitat under this scenario. Further, the increase of 20.3% and 3.3% in a moderately and optimum suitable area, respectively, were found to occur under SSP126 (2070) (Fig. 5). About 20.9% reductions in the highly suitable area was projected under SSP126 (2070). These are predicted mainly in eastern Maharashtra, Chhattisgarh, Bihar, Jharkhand and Telangana states of India.

Under the scenarios SSP126 (2050) and SSP126 (2070), the model showed that certain areas that are presently low and moderately suitable for the habitat of *T. tabaci* might become highly suitable for future potential distribution. Central and Southern states and some parts of northern India are classified as highly suitable for *T. tabaci* under the SSP126 scenario in 2050 and 2070.

The suitable area under SSP585 for 2050 and 2070 were 2.49 × 10^6^ km^2^ and 2.47 × 10^6^ km^2^, accounting for 77.3% and 76.8%, respectively, of the land area of India (Table 2). The highly, optimum, moderately and low suitable habitable area for *T. tabaci* distribution under SSP585 (2050) was 9.47 × 10^5^ km^2^, 7.54 × 10^5^ km^2^, 4.85 × 10^5^ km^2^, and 3.07 × 10^5^ km^2^, respectively. About 7.38 × 10^5^ km^2^ area was projected as unsuitable under SSP585 (2050).

The model predicted an increase of low suitability (35.8%) under a high emission scenario, SSP585 (2050), compared to current conditions (Fig. 5). The moderate and optimum suitable area was found to increase by 21.3% and 1.3%, respectively compared to the current condition. A 24.2% and 51.7% reduction in highly suitable habitats were projected under SSP585 (2050) and SSP585 (2070), respectively. The highly, optimum, moderately and low suitable area for 2070 under SSP585 was 7.73 × 10^5^ km^2^, 7.54 × 10^5^ km^2^, 5.80 × 10^5^ km^2^ and 3.67 × 10^5^ km^2^, respectively (Table 2). Likewise, the model projected a 46.5% increase in low-suitability habitat areas and a 2.6% increase in unsuitability areas under SSP585 (2070). Amongst habitat classes, the highly suitable habitat for *T. tabaci* is predicted to contract by 24.2% and 51.7% under SSP126 and SSP585, respectively (Fig. 5). These were mainly in Jharkhand, Bihar, Chhattisgarh, eastern Maharashtra and Telangana states of India. Overall, under SSP126 and SSP585, the bioclimatic suitability for *T. tabaci* is projected to turn down in India's central and southern states, which are currently highly suitable. Moreover, under both scenarios, high and optimum suitability habitat areas are predicted to increase in some of the northern states located at high latitudes.

The multi-model prediction of suitable area for *T. tabaci* according to BCC-CSM2-MR, CanESM5, CNRM-CM6-1 and MIROC6 climate data under low SSP126 and high emission SSP585 scenarios were given in Supplementary table 3. The analysis of the prediction of BCC-CSM2-MR under low emission (SSP126) and high emission (SSP585) showed that the maximum highly suitable area for *T. tabaci* attained under SSP585 (2050) than the SSP126. However, under SSP585 (2070), it tends to contract mainly in central India, and an increase of highly suitable areas is predicted mainly in Jharkhand and Bihar states of India. The optimum suitability found to decrease under SSP585 (2050), with a slight increase in 2070, mainly in western Maharashtra. Conversely, the prediction of the CanESM5 model under different SSPs revealed that the highly suitable area attained maximum under SSP126 (2070), and the minimum under SSP585, which was the same as the prediction of the BCC-CSM2-MR model. In contrast, the optimum suitable area reached the maximum under SSP126 (2050).

Likewise, the prediction of CNRM-CM6-1 shows that the highly suitable area attained maximum under SSP126 (2050) and SSP585 (2050), whereas much loss of suitability area likely under SSP585 (2070). Similarly, in the prediction of MIROC6, the maximum highly suitable area was attained under low-forcing SSP126 (2050), with a marginal reduction in high-forcing SSP585 (2070). The decrease in high suitability is projected mainly in Maharashtra and Telangana states of India.

The prediction of all the four models revealed that under a high forcing scenario, the highly suitable habitat for *T. tabaci* tends to decrease marginally (Supplementary table 4). The prediction of the BCC-CSM2-MR model under a low-forcing scenario revealed that there would be a loss of 1.27 × 10^5^ km^2^ and 1.66 × 10^5^ km^2^ area under SSP126 (2050) and SSP126 (2070), respectively. Likewise 8.28 × 10^4^ km^2^ and 2.93 × 10^5^ km^2^ area loss predicted in highly suitable area under SSP585 (2050) and SSP585 (2070), respectively. The prediction of the CanESM5 model revealed a maximum (5.43 × 10^5^ km^2^) area decrease in highly suitable areas under SSP585 (2070), with a minimal reduction under SSP126 (2070). Under both low and high-emission scenarios, the highly suitable area is predicted to be a contract, while the medium suitability area is predicted to increase. The prediction of the CNRM-CM6-1 model revealed that the maximum reduction of highly suitable area 3.89 × 10^5^ km^2^ attained under SSP585 (2070). In contrast, the optimum suitability habitat for *T. tabaci* is projected to increase under both low and high-forcing scenarios. The prediction of the MIROC6 model under SSP126 revealed 1.31 × 10^5^ km^2^ and 1.93 × 10^5^ km^2^ area would lost under SSP126 (2050) and SSP126 (2070), respectively. Likewise, a decrease of 2.44 × 10^5^ km^2^ and 3.77 × 10^5^ km^2^ of the highly suitable area was predicted under SSP585 (2050) and SSP585 (2070), respectively. Moreover, the optimum suitability area would increase under low and high forcing scenarios.

## Discussion

Climate modelling for habitat suitability has unequivocally proven that climate change will significantly impact crop pests' distribution^3–5^. Despite the errors and uncertainties in the outputs of species distribution modelling^59^, SDM is still considered an effective tool to predict future changes in the distribution of a species^54,60^. Studies attributed that temperature and precipitation affects the species distribution, survival, and development of *T. tabaci* and other pest species^61–65^. The current study estimated the potential geographic distribution of *T. tabaci* in India by analyzing the current CMIP6 data with greater prediction accuracy. *T. tabaci* has a wide distribution range in India, spread across central, southern, north and northeastern states. Based on a large scale climate data, current model projected its geographical distribution in India to 1.17 × 10^6^ km^2^ as the highly suitable habitat for the species under current climatic conditions, in which the center areas are in Maharashtra, Gujarat, Madhya Pradesh, the southern states of Andhra Pradesh, Telangana, the northern states of Uttar Pradesh, Bihar, Jharkhand, Chhattisgarh, Delhi, Haryana, Punjab and Uttarakhand, and northeastern states like Sikkim, Assam, Meghalaya and Tripura. This model exactly reflects the current distribution of *T. tabaci* in India and in agreement with the occurrence data. MaxEnt utilizes continuous and categorical data, incorporates interactions between different variables, and predicts and avoids commission errors^66,67^.

Model results showed that Annual Mean Temperature (bio1) and two precipitation variables (bio12, bio15) were among the most important bioclimatic variables, which contributed 68.3% to the current distribution. Previous studies on growth and distribution of *T. tabaci* showed that temperature and precipitation are significant determinants for reproduction, development, migration and dispersal of this species^24,68^. Further, temperature-induced reproductive quiescence is also evident in adult thrips^63^. Likewise, we found that bio1 (33.3%) and bio12 (19.6%) were the most important factors for *T. tabaci* and with the suitable range of 22–28 °C and 300–1000 mm, respectively for its distribution. The changes in the precipitation pattern under climate change may directly or indirectly impact pest survival. The warming temperatures are attributed affecting the development rate, survival, metabolic rate, and number generation of insects^65^. Moreover, smaller size insects like *T. tabaci* are further vulnerable to heavy precipitation because it washes off them from its hosts^69^. Hard precipitation reduces the thrips damage on plants^56^; detrimental to thrips larvae^31^ and suppresses adult dispersal^70^. Warm temperature affects insect population growth by reducing cold-related mortality^71^ and shortening their generation times. A temperature of 30 °C was optimum for *T. tabaci* growth and development and temperature rises from 25 to 35 °C shortens the development period, total life cycle and per cent survival rate of *T. tabaci*. Bergant et al. assessed the potential impact of climate change on the development dynamics of *T. tabaci* using Global Circulation Models (GCMs). They suggested that the expected temperature increase will lead to a larger number of degree days, resulting in increased generations and consequently more crop damage^27^. Higher precipitation (11.2 mm) and daily mean temperature < 10 °C reduced the *T. tabaci* movement, and daily mean temperature beyond 14.4 °C favoured population build-up^61^. The aerial dispersal of adult *T. tabaci* increased when the temperature raised beyond 17 °C, and 90% of the aerial dispersion was between 20.8 and 27.7 °C and no dispersal when the temperature was > 30.6 °C^72^. Studies also reported the prevalence of diverse response of *T. tabaci* to temperature changes^26,73^. This suggests that survival and distribution of *T. tabaci* be directly affected mainly by temperature and precipitation.

MaxEnt predicted that under the current habitat, the high-suitability habitats identified overlap with India's main onion-growing states where *T. tabaci* recorded at moderate to high densities^74^. Therefore, MaxEnt was reliable in predicting *T. tabaci* distribution, and the current prediction aligns with the recent reports.

The model shows that, under future climatic change SSP126 (2050), SSP126 (2070) and SSP585 (2050) scenarios, the highly suitable areas are concentrated in Gujarat, southern Rajasthan, Maharashtra, Karnataka and Andhra Pradesh. Besides, some of the northern states including Uttar Pradesh, Punjab, Haryana, Uttarakhand, and Himachal Pradesh, are highly suitable in this scenario. However, under high emission scenario SSP585 (2070), there was a reduction in highly suitable habitat areas compared to current SSP126 (2050), SSP126 (2070) and SSP585 (2050). Moreover, the model predicted an increase of optimum suitability areas in all these scenarios. The areas under moderate suitability in the current climatic condition are projected to optimum suitability in both high and low-emission scenarios. A mechanistic niche model (CLIMAX) by Park et al. for a thrips species *Thrips plami* in Korea stated that the geographical distribution of polyphagous *T. palmi* could be easily expanded to regions wherever the host exists^75^. Maximum Temperature of the Coldest Month (bio6) and the Maximum Temperature of the Warmest Month were the highest contributing variables (82.5% to the model) determining the potential distribution of *T. palmi* in Korea^76^. Therefore, winter temperature would be the most influencing factor that can increase the size of overwintering population during the crop growing season. The net reproductive rate of *T. palmi* reaches its maximum of around 25 °C, and the generation time is 25 days^25^. Application of MaxEnt by Shogren and Paine for predicting the invasive potential of another thrips species, *Klambothrips myopori* revealed that temperature seasonality was a major variable contributing (64.9%) to the model^77^. MaxEnt projections recovered the invasive range in California, but the known native range of *K. myopori* in Australia could not recovered enough. Precipitation of the Wettest Month (bio16), Temperature Annual Range (bio7), Maximum Temperature of the Warmest Month (bio5), as well as Precipitation of Warmest Quarters (bio18), were the most significant predictors of legume flower thrips, *Megalurothrips sjostedti* habitat distribution^78^. The success of species distribution depends on many factors, not only climatic. The factors such as land cover, land use, landscape structures and dispersal success need to be considered for prediction as they may seriously impact species distribution^76,79^. The season and cropping system determine the population structure of *T. tabaci* and has been attributed to variations in colonization patterns in response to cropping systems or strong establishment of particular genotypes on particular hosts^80^.

When the predictions of different models were compared under the scenarios SSP126 and SSP585, it was found that the amount of suitable habitable area gained and lost in each model under the same scenario varied. For example, the CanESM5 in the SSP585 model predicted that the area of the optimum suitable area of *T. tabaci* would decrease in the future, but the CNRM-CM6-1 predicted that it would gain. Moreover, in both scenarios, the prediction results of all four models consistently shift the highly suitable area, moderately suitable area, and low suitable area (gain or loss simultaneously). In the vast majority of cases, all four models revealed a unified trend in their predicting area suitable for *T. tabaci* in most cases. It illustrates that multi-model predictions could still help avoid uncertainty or display the phenomenon that is more likely to happen. While compared to the low-forcing scenario SSP126, the prediction of high-forcing scenario SSP585 lost the most highly suitable area. Conversely, the highly suitable region would degenerate, expanding the optimum and moderately suitable areas.

This is the first study in India that predicted the potential geographical distribution of *T. tabaci* under climate change scenarios using MaxEnt. The projection revealed that decrease in habitat suitability in the region, where the *T. tabaci* distributions were concentrated. Further, few pockets of higher latitudes are projected to be highly and optimistically suitable area under future climatic scenarios. The climate change related with latitudinal and altitudinal shift in species distribution, generally migrates to higher elevations and latitudes as climates warm. Our projection revealed that the suitability areas centered among major onion-growing central and southern states of India remain as potentially habitable areas for *T. tabaci* distribution in the near future also. Further, consistently, current analysis shows that under both the low (SSP126) and (SSP585) greenhouse emission scenarios the suitable habitat concentrates in higher latitudes states like Uttar Pradesh and Uttarakhand. Low-latitudes regions including central and southern regions, such as Maharashtra, Karnataka, Telangana and Andhra Pradesh states would see a decrease in distribution. While considering cultivation area expansion^81^ to fulfill the domestic and export market, the potential future distribution, especially states having high habitat suitability for *T. tabaci* should frame sound management strategies to lower the pest pressure and prevent economic damage. The policies should ensure the appropriate monitoring and management strategy to limit pest outbreaks in these areas. Although the habitat predicted by the MaxEnt model in this study was remarkable, the limited number of occurrence coordinates, size of the study area, and choice of predictor variable all carry the risk of errors and ambiguity. MaxEnt is an ecological niche model that does not consider the influence of biotic factors, tri-trophic interaction (plant-pest predators and parasitoids), and management strategies that could significantly impact the species distribution^82^. However, it may be assumed that model will also perform well in future climatic scenarios considering how well MaxEnt performed in the current habitat. For a deeper understanding of *T. tabaci* survival, studies on host phenology, off-season survival, and dispersal behavior are also essential.

## Conclusions

The current study performed detailed analysis on the suitable habitat of *T. tabaci* in India under current and future climate change scenarios, SSP126 and SSP585, which can serve as an important step in developing strategies and policies for effective management of *T. tabaci* in onion. MaxEnt projected that bio1, bio12, and bio15 are the important bioclimatic variables, which greatly impacted the habitat suitability of *T. tabaci,* with the suitable range of 22–28 °C; 300–1000 mm and 70–160, respectively. This suggests that annual mean temperature (> 30.0 °C), annual mean rainfall (> 1000 mm) and precipitation seasonality (> 160) under climate warming would contract the species distribution in low-latitude regions, mainly central and southern states of India. Moreover, habitat suitability in few pockets of northern Indian states would concentrate further increase the optimum suitable habitat. The prediction of the BCC-CSM2-MR, CanESM5, CNRM-CM6-1 and MIROC6 model, suggests that the highly suitable area for *T. tabaci* would likely to contract under both SSP126 and SSP585. The findings of this study could aid researchers in better understanding the species distribution and a theoretical reference for the identification of potential areas for *T. tabaci* in India. This will help in devising effective pest management strategies under climate change in the future.

## Supplementary Information

Supplementary Tables.

## Data Availability

On request, data can be obtained from the corresponding author.
