# Supplementary material for: Predicting the potential geographical distribution of onion thrips, Thrips tabaci in India based on climate change projections using MaxEnt
Source: Sci Rep. 2023 May 16;13:7934. doi: 10.1038/s41598-023-35012-y (PMC10188569; doi:10.1038/s41598-023-35012-y)
Supplement: Supplementary file 1 — Supplementary Tables. [file 41598_2023_35012_MOESM1_ESM.pdf]

Predicting the potential geographical distribution of onion thrips, *Thrips tabaci* in India based on climate change projections using MaxEnt

V. Karuppaiah[1]*, R. Maruthadurai[2]*, Bappa Das[2], P.S. Soumia[1], Ankush S. Gadge[1], A. Thangasamy[1], S.V. Ramesh[3], Dhananjay V. Shirsat[1], V. Mahajan[1], Hare Krishna[4] and Major Singh[1]

[1] ICAR-Directorate of Onion and Garlic Research, Rajgurunagar, Pune 410 505, Maharashtra, India;
[2] ICAR-Central Coastal Agricultural Research Institute, Ela, Old Goa 403 402, Goa, India;
[3] ICAR-Central Plantation Crops Research Institute, Kasaragod 671 124, Kerala, India;
[4] ICAR-Indian Institute of Vegetable Research, Varanasi 221 305, India;
*****Corresponding author(s): karuppaiahv2008@gmail.com (V.K); duraiento@gmail.com (R.M); Tel.: (+91-02135 222026)

S1. Species occurrence coordinates

	Thrips tabaci	
	**Latitude**	**Longitude**
Thrips tabaci	22.975084	88.434509
Thrips tabaci	21.2514	81.6296
Thrips tabaci	13.132068	77.489234
Thrips tabaci	19.997454	73.789803
Thrips tabaci	29.594189	79.653898
Thrips tabaci	20.707228	77.00296
Thrips tabaci	29.685629	76.990547
Thrips tabaci	29.45598412	79.6565399
Thrips tabaci	24.587999	76.157578
Thrips tabaci	34.112799	74.793771
Thrips tabaci	13.92993	75.5081
Thrips tabaci	16.1634513	75.6172122
Thrips tabaci	32.1023191	76.5581075
Thrips tabaci	24.063001	75.087733
Thrips tabaci	18.8427	73.8838
Thrips tabaci	34.083656	74.797371
Thrips tabaci	30.900971	75.8572698
Thrips tabaci	26.49115	80.307251
Thrips tabaci	21.3266	71.0253
Thrips tabaci	26.8505899	75.7909157
Thrips tabaci	23.2307	79.5927
Thrips tabaci	25.853509	85.779114
Thrips tabaci	21.33595	83.93517
Thrips tabaci	13.0782	77.5792
Thrips tabaci	10.0854	77.6509
Thrips tabaci	25.6888	91.9179
Thrips tabaci	30.9717068	76.1080752
Thrips tabaci	30.900975	75.8572658
Thrips tabaci	30.900965	75.857277
Thrips tabaci	31.581839	75.889659
Thrips tabaci	31.57325	75.8915
Thrips tabaci	23.97301449	91.37834061
Thrips tabaci	23.5539166	91.4682362
Thrips tabaci	22.988559	91.66317

Thrips tabaci	24.075719	91.605318
Thrips tabaci	23.906435	91.313186
Thrips tabaci	23.82737	91.43199
Thrips tabaci	21.75518	70.62043
Thrips tabaci	22.70249	71.72044
Thrips tabaci	21.63484	70.39771
Thrips tabaci	21.554853	70.703468
Thrips tabaci	21.0955	70.15201
Thrips tabaci	21.933307	69.783293
Thrips tabaci	19.112316	73.730133
Thrips tabaci	18.8226	74.104
Thrips tabaci	18.87	74.0212
Thrips tabaci	18.8311	73.9808
Thrips tabaci	19.1178	73.9741
Thrips tabaci	19.265451	73.981766
Thrips tabaci	19.1770314	74.1103155
Thrips tabaci	20.63032	78.47011
Thrips tabaci	18.459289	74.587335
Thrips tabaci	17.9743351	74.4247094
Thrips tabaci	14.972417	75.335569
Thrips tabaci	14.623801	75.621788
Thrips tabaci	14.631413	75.406903
Thrips tabaci	15.7214	75.3849
Thrips tabaci	15.231288	75.577157
Thrips tabaci	15.4315	75.6355
Thrips tabaci	15.8133	74.8566
Thrips tabaci	15.7522	75.1253
Thrips tabaci	15.2553	75.2475
Thrips tabaci	15.5605	75.3556
Thrips tabaci	15.371598	75.10116
Thrips tabaci	11.41	76.699997
Thrips tabaci	11.110695	77.348045
Thrips tabaci	10.7905	78.7047
Thrips tabaci	11.342423	77.728165
Thrips tabaci	11.23	78.879997
Thrips tabaci	10.365581	77.970657
Thrips tabaci	20.0280604	73.9709312
Thrips tabaci	20.0698	74.0462
Thrips tabaci	20.079966	74.109314

Thrips tabaci	18.5320397	74.0314741
Thrips tabaci	20.105982	74.235542
Thrips tabaci	20.080114	74109818
Thrips tabaci	20.4174	74.41955
Thrips tabaci	19.50724	74.55072
Thrips tabaci	19.85306	74.000633
Thrips tabaci	19.8785	73.9705
Thrips tabaci	20.4736	74.1871
Thrips tabaci	19.5761	74.207
Thrips tabaci	18.751123	74.636131
Thrips tabaci	27.703669	76.201195
Thrips tabaci	28.63701	77.15044
Thrips tabaci	18.8402815	78.9505623
Thrips tabaci	29.151869	75.71581
Thrips tabaci	30.9098889	74.1386627
Thrips tabaci	30.537	74.9873
Thrips tabaci	30.517947	75.8887422
Thrips tabaci	30.42005	75.73295
Thrips tabaci	30.87905	75.8418499
Thrips tabaci	26.1572572	80.0772302
Thrips tabaci	23.54335812	74.42980782
Thrips tabaci	27.6321	76.7106
Thrips tabaci	27.862247	76.550146
Thrips tabaci	27.8133	76.6948
Thrips tabaci	26.668365	87.430496
Thrips tabaci	23.993964	91.558566
Thrips tabaci	24.012594	91.835487
Thrips tabaci	33.8918	74.9811
Thrips tabaci	30.86	77.173
Thrips tabaci	27.8974	78.088
Thrips tabaci	27.710951	77.937683
Thrips tabaci	28.0491896	77.9610158
Thrips tabaci	27.939196	77.842445
Thrips tabaci	28.043842	77.577423
Thrips tabaci	19.2608	76.7748
Thrips tabaci	32.041943	75.405334
Thrips tabaci	31.22402	75.770798
Thrips tabaci	26.5243	89.1075
Thrips tabaci	10.0676	78.0488

Thrips tabaci	22.7877	75.71913
Thrips tabaci	23.4186	76.266701
Thrips tabaci	24.597349	76.16095
Thrips tabaci	13.1365	75.6403
Thrips tabaci	11.129392	78.8336849
Thrips tabaci	10.5166293	78.0620541
Thrips tabaci	11.1282	78.8391
Thrips tabaci	11.147227	78.5983889
Thrips tabaci	11.2015986	78.7957279
Thrips tabaci	11.14723	78.5983859
Thrips tabaci	11.9261	76.9437
Thrips tabaci	11.8083	76.6927
Thrips tabaci	12.489398	78.567907

S2. List of bioclimatic variables used for this study

Variables	Abbreviation	Units
Annual Mean Temperature	bio1	°C
Mean Diurnal Range (Mean of monthly (Tmax-Tmin))	bio2	°C
Isothermality (bio2/bio7) (*100)	bio3	-
Temperature Seasonality (standard deviation*100)	bio4	°C
Max Temperature of Warmest Month	bio5	°C
Min Temperature of Coldest Month	bio6	°C
Temperature Annual Range (Bio_5-Bio_6)	bio7	°C
Mean Temperature of Wettest Quarter	bio8	°C
Mean Temperature of Driest Quarter	bio9	°C
Mean Temperature of Warmest Quarter	bio10	°C
Mean Temperature of Coldest Quarter	bio11	°C
Annual Precipitation	bio12	mm
Precipitation of Wettest Month	bio13	mm
Precipitation of Driest Month	bio14	mm
Precipitation Seasonality (Coefficient of Variation)	bio15	Fraction
Precipitation of Wettest Quarter	bio16	mm
Precipitation of Driest Quater	bio17	mm
Precipitation of Warmest Quarter	bio18	mm
Precipitation of Coldest Quarter	bio19	mm

Bioclimatic variables are biologically meaningful indicators that describe how climate affects ecosystems and services. They are derived from monthly temperature and rainfall values that then represent annual and seasonal climatic trends.

Supplementary Table 3. Area (km^2) under various classes for BCC-CSM2-MR, CanESM5, CNRM-CM6-1, MIROC6 models during 2050 and 2070 under low (SSP126) and high (SSP585) emissions scenario

Class	SSP 126							
	BCC-CSM2-MR		CanESM5		CNRM-CM6-1		MIROC6	
	2050	2070	2050	2070	2050	2070	2050	2070
Unsuitable	7.41×10^5	7.47×10^5	7.25×10^5	7.07×10^5	7.37×10^5	7.33×10^5	7.55×10^5	7.62×10^5
Low	2.47×10^5	2.45×10^5	3.83×10^5	3.81×10^5	2.50×10^5	2.43×10^5	2.20×10^5	2.26×10^5
Medium	4.46×10^5	4.44×10^5	4.88×10^5	5.08×10^5	4.31×10^5	4.63×10^5	4.12×10^5	4.96×10^5
Optimum	7.52×10^5	7.89×10^5	7.84×10^5	7.31×10^5	7.45×10^5	7.93×10^5	8.02×10^5	7.67×10^5
High	1.04×10^6	1.00×10^6	8.52×10^5	9.05×10^5	1.06×10^6	1.00×10^6	1.04×10^6	9.81×10^5
	SSP 585							
Unsuitable	7.50×10^5	7.60×10^5	6.91×10^5	7.51×10^5	7.47×10^5	7.54×10^5	7.61×10^5	7.66×10^5
Low	2.43×10^5	2.60×10^5	4.45×10^5	5.51×10^5	2.75×10^5	3.28×10^5	2.64×10^5	3.30×10^5
Medium	4.28×10^5	5.37×10^5	6.40×10^5	7.36×10^5	4.26×10^5	4.96×10^5	4.47×10^5	5.51×10^5
Optimum	7.18×10^5	7.94×10^5	6.67×10^5	5.62×10^5	8.03×10^5	8.70×10^5	8.29×10^5	7.88×10^5
High	1.09×10^6	8.81×10^5	7.87×10^5	6.31×10^5	9.81×10^5	7.85×10^5	9.30×10^5	7.96×10^5

Supplementary Table 4. The future gain and loss of suitable habitat area (km^2) under low (SSP126) and high (SSP585) emissions scenario

Class	SSP 126							
	BCC-CSM2-MR		CanESM5		CNRM-CM6-1		MIROC6	
	2050	2070	2050	2070	2050	2070	2050	2070
Unsuitable	2.10×10^{3}	7.94×10^{3}	-1.35×10^{4}	-3.21×10^{4}	-1.46×10^{3}	-6.08×10^{3}	1.59×10^{4}	2.25×10^{4}
Low	5.12×10^{4}	4.93×10^{4}	1.87×10^{5}	1.85×10^{5}	5.42×10^{4}	4.75×10^{4}	2.45×10^{4}	3.02×10^{4}
Medium	6.70×10^{4}	6.50×10^{4}	1.09×10^{5}	1.29×10^{5}	5.21×10^{4}	8.43×10^{4}	3.27×10^{4}	1.17×10^{5}
Optimum	7.36×10^{3}	4.45×10^{4}	3.93×10^{4}	-1.3×10^{5}	3.86×10^{2}	4.84×10^{4}	5.77×10^{4}	2.27×10^{4}
High	-1.27×10^{5}	-1.66×10^{5}	-3.22×10^{5}	-2.69×10^{5}	-1.05×10^{5}	-1.74×10^{5}	-1.31×10^{5}	-1.93×10^{5}
	SSP 585							
Unsuitable	1.15×10^{4}	2.07×10^{4}	-4.76×10^{4}	1.25×10^{4}	8.44×10^{3}	1.45×10^{4}	2.22×10^{4}	2.74×10^{4}
Low	4.79×10^{4}	6.41×10^{4}	2.50×10^{5}	3.55×10^{5}	7.94×10^{4}	1.32×10^{5}	6.85×10^{4}	1.34×10^{5}
Medium	4.97×10^{4}	1.58×10^{5}	2.61×10^{5}	3.56×10^{5}	4.68×10^{4}	1.17×10^{5}	6.85×10^{4}	1.72×10^{5}
Optimum	-2.63×10^{4}	4.98×10^{4}	-7.70×10^{4}	-1.82×10^{5}	5.83×10^{4}	1.25×10^{5}	8.48×10^{4}	4.36×10^{4}
High	-8.28×10^{4}	-2.93×10^{5}	-3.86×10^{5}	-5.43×10^{5}	-1.92×10^{5}	-3.89×10^{5}	-2.44×10^{5}	-3.77×10^{5}
